# Binder-Free Direct Ink Writing of a Concentrated Dispersion of One-Dimensional Lepidocrocite Titanate Nanofilaments

**DOI:** 10.1021/acsnanoscienceau.5c00110

**Published:** 2025-11-18

**Authors:** Francis Mekunye, Adam D. Walter, Gregory R. Schwenk, Michel W. Barsoum, Virginia A. Davis

**Affiliations:** 1 Department of Chemical Engineering, 1383 Auburn University, 212 Ross Hall, Auburn, Alabama 36830, United States; 2 Department of Materials Science and Engineering, 6527 Drexel University, 3141 Chestnut St, Philadelphia, Pennsylvania 19104, United States

**Keywords:** direct ink writing, lepidocrocite titanate, yield stress, liquid crystal, nematic

## Abstract

Direct ink writing (DIW) of nanomaterial dispersions enables the production of structures and devices that combine the benefits of the nanomaterial properties with use-specific manufacturing designs. However, printing nanomaterial inks requires the ability to produce stable dispersions at concentrations high enough to meet the rheological criteria for DIW. Herein, we report on DIW of one-dimensional lepidocrocite (1DL) nanofilament, NF, dispersions at concentrations of 150 g/L, which are much greater than previously achieved concentrations. Moreover, the resulting birefringent, binder-free nematic gel can be printed into standard test patterns with outstanding dimensional accuracy, structural integrity, and shape fidelity. Both the ability to directly produce higher concentrations and the ability to print them open new opportunities for the production of functional 1DL materials and devices.

## Graphical abstract

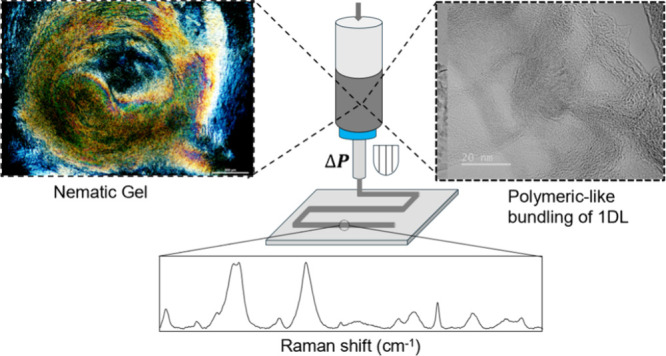

## Introduction

Additive manufacturing of soft materials is an emerging technology with expanding applications in electronics, catalysis,^1^ biomedical engineering, and energy storage,^2–4^ especially for ceramics such as titania (TiO_2_). Additive manufacturing methods, including powder-based, bulk solid-based, and slurry-based methods, have revolutionized the production of ceramic materials.^5,6^ Among ceramic additive manufacturing methods, direct ink writing (DIW) is viewed as the most adaptable since it requires lower-cost equipment and can theoretically be used to print nearly any material. However, DIW requires creation of a stable dispersion, or ink, at sufficiently high concentrations for the ink to be controllably extruded out of the printer nozzle and hold its shape after deposition. The high densities and attractive interactions between ceramic particles can make this a challenge. Many ink formulations for these applications rely on the use of polymeric^7,8^ or composite^9^ binders to achieve the rheological properties, such as yield stress and viscoelasticity, required for printing and enhancing ink stability. Alongside advancements in ceramic manufacturing methods, new ceramic materials have continued to be discovered. An exciting example is one-dimensional lepidocrocite titanate nanofilaments (1DLs).^10^ Compositionally similar to titania, lepidocrocite titanates (LTs)^11^ are oxygen-rich layered materials comprised of edge-sharing TiO_6_ octahedra that require cations to exist between the layers for charge balance.^12–14^ Like titania, LTs can be used for a variety of applications such as dye-sensitized solar cells,^15^ batteries,^16^ composite catalyst supports,^17^ or directly as photocatalysts.^18^ This, by no means, is an exhaustive list of applications for these versatile nanomaterials.

In the LT literature, the narrowest c-dimension is ≈10 nm,^19^ achieved through complex processing. In many other studies, the c-dimension is significantly wider, which, in some cases, results in tubular morphologies.^12–14^ 1DLs differ from LTs by their reduced dimension along the *c*-axis (Figure 1A), making them truly one-dimensional from a quantum confinement standpoint, as evidenced by their high band-gap energies, ≈4 eV. The dimensions are determined by synthesis conditions; for example, when dried, 1DLs self-assemble along the *c*-direction into ribbons approximately 3–5 nm wide unless this process is arrested through polymer wrapping, as shown in the transmission electron microscope (TEM) micrographs in Figure S1.^20,21^ Diluting the colloidal suspension can assist in retarding this assembly and can also be used to control the band gap energy of the resulting solid.^22^ This assembly can also be controlled by modifying the solvent system of the dispersion.^23^

**Figure 1:**
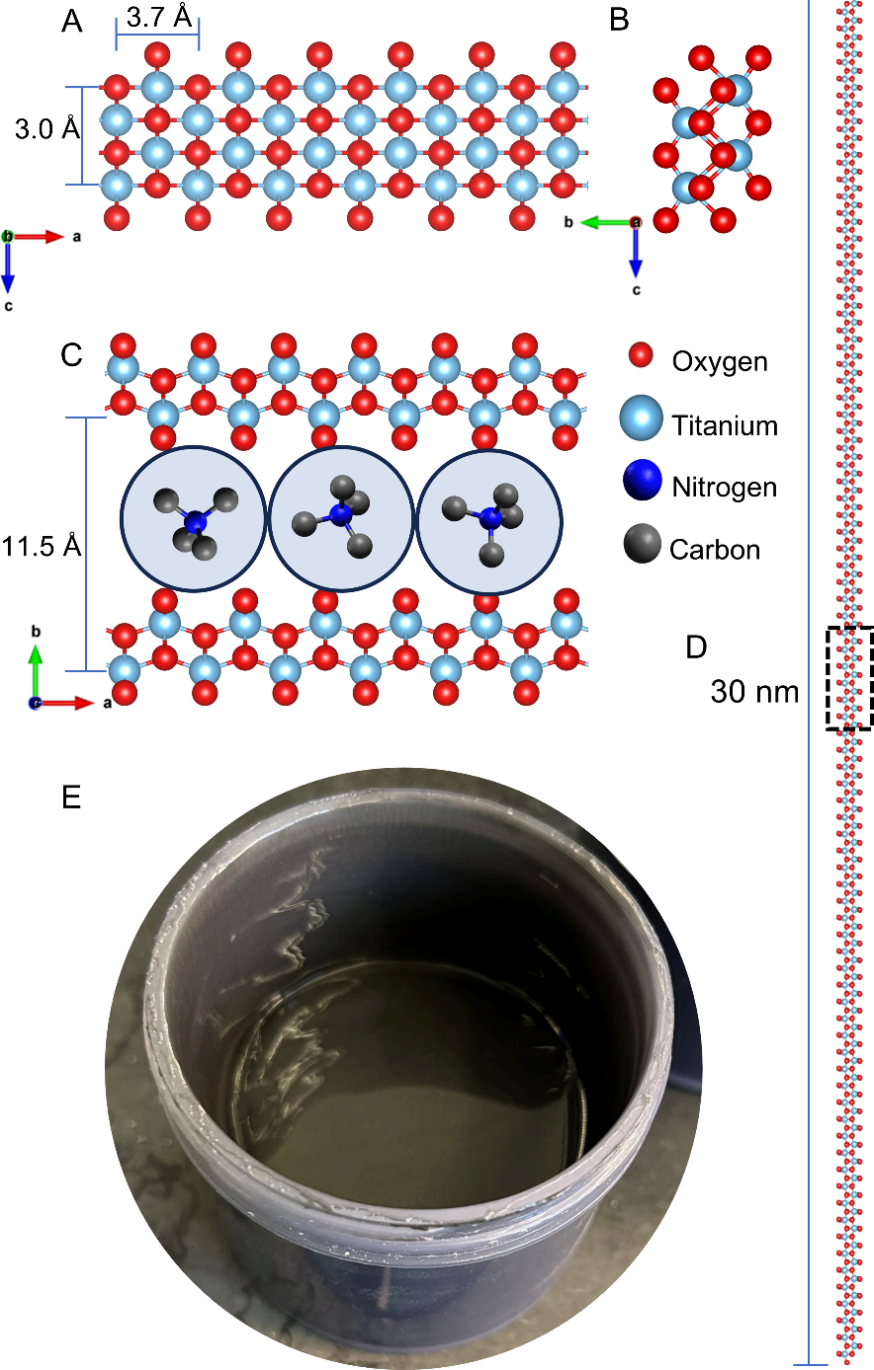
Structure of 1DLs and 1DL ink. (A) a-c plane, (B) b-c plane, and (C) a-b plane of the 1DL base unit. Stacking shown in (C) is ABA stacking with TMA^+^ cations in between the layers. Blue circles refer to hydration shells of each TMA^+^ obtained from molecular dynamics calculations.^24^ Reproduced from ref 23. (D) a-b plane of a single 1DL filament; dashed box corresponds to area shown in (C) showcasing its polymer-like aspect ratio. Copyright 2024 American Chemical Society. (E) Photograph of ≈100 mL of 1DL ink in a high shear mixing cup.

Notably, producing 1DLs is remarkably simple, reacting a diverse collection of Ti-based precursors such as carbides, borides, nitrides, oxysulfates, etc., with tetramethylammonium hydroxide (TMAOH) in polyethylene bottles, at ambient pressures and temperatures <100 °C. In this work, titanium diboride (TiB_2_) was used as the Ti-based precursor, and its X-ray diffraction (XRD) pattern is presented in Figure S2. During this reaction, 1DLs grow along the *a*-direction (Figure 1A,C), while being templated by the structure-directing base TMA^+^ ^25^ during formation in the *b-* and *c-*directions (Figure 1B,C). According to a more detailed TEM study, 1DLs generally exist as bundles of ≈30 nm long filaments (Figure 1D).^26^ (A much more detailed explication of the 1DL structures and their self-assembly can be found in refs 21 and 38). When alkali hydroxides are used instead of TMAOH, phases similar to the alkali LTs reported in literature^13,14,16,27^ are obtained.^28^

Leveraging their remarkably enhanced surface areas, 1DLs have demonstrated efficient ion exchangeability among organic and inorganic cations and even sensitization effects by common textile dyes.^29–32^ Additionally, they have been investigated for use as a sulfur host in lithium–sulfur batteries,^33^ among other applications.^10,34–36^

One of the major benefits of 1DLs is how simple it is to create stable, nonagglomerating aqueous dispersions. Until the work detailed in this letter, 1DL dispersions of uncontrolled concentrations have been easily synthesized. These were formed by adding water to damp ethanol-washed TiB_2_-derived 1DL sediment. The sediment is comprised solely of micrometer-scale 1DL porous mesoscale particles (PMPs) that are damp with ethanol, EtOH. Until water is added to this sediment, the particles remain whole, act as a biphasic system with EtOH, and are not considered a dispersion. The water breaks up the damp particles and suspends them into their primary 5 × 7 Å^2^ NFs. The resulting dispersion is basic (pH ≈ 10) and remains shelf-stable for months. Due to their relatively low concentrations (maximum ≈ 40 g/L), these 1DL aqueous dispersions are not viscous enough for DIW or other 3D printing processing methods.

In an effort to find the limit of 1DL suspensions and develop a highly viscous 1DL aqueous dispersion for DIW, a systematic study was carried out (Table 1). Adding a known mass of sediment, assuming it is ≈38 ± 2 wt % 1DL with the balance being EtOH confined within the 1DL pores (determined by comparative mass assessment after drying the centrifuged sediment at 80 °C in air for 12 h), to a certain mass of water in 50 mL centrifuge tubes allowed for an approximate calculation for the final 1DL concentrations. After mixing via vortex shaking, or by high shear mixing when the viscosity is too great for vortexing which is the case for the two highest loadings reported, the dispersion is centrifuged to separate the unreacted precursor and nonsuspended 1DLs. The colloidal dispersion is then carefully poured off, leaving the waste—in the form of any non-1DL component (i.e., unreacted precursor)—behind as lost mass. The final 1DL concentration is then determined by drying a known volume of dispersion and weighing the final solid (after vacuum filtration and air drying at 80 °C for 12 h). Interestingly, across the entire range tested, the mass loss was 18 ± 3 wt %, and in all cases, the colloidal suspensions did not contain residual TiB_2_. This suggests that centrifuging the dispersions, even at high concentrations, is effective in removing the unreacted precursor if present. It must be mentioned that there is a sizable amount of EtOH in the concentrated dispersions (≈30 wt % in the most concentrated case). In summary, by simply reducing the amount of water added to the reaction product in the form of a sediment, a highly viscous and concentrated (≈150 g/L) 1DL dispersion suitable for DIW, can be directly synthesized, without any further processing or additives.

**Table 1:** Summary of 1DL Dispersion Synthesis Conditions^a^

ethanol damp sediment (g)	water (g)	1DL (g)	1DL/water mass ratio	approx. volume (mL)	calculated [1DL] (g/L)	final [1DL] (g/L)	mass loss
0.5	40	0.19	0.005	40.5	5	3.9	17%
1	40	0.38	0.01	41	9	7.88	15%
2	40	0.76	0.02	42	18	16	12%
4	40	1.52	0.04	44	35	29.3	15%
6	40	2.28	0.06	46	50	41	17%
8	40	3.04	0.08	48	63	50.4	20%
10	40	3.8	0.10	50	76	61.1	20%
15	30	5.7	0.19	45	127	102.5	19%
20	20	7.6	0.38	40	190	146.5	23%

a Columns 1 and 2 represent the amounts of damp sediment and water added. Columns 3 and 4 represent relevant 1DL masses and ratios, calculated from 1 and 2, assuming 38 wt % of the sediment is 1DL solid and the balance is EtOH. Column 5 represents total volume of the colloid and is approximated using the indications on the centrifuge tube. Column 6 represents the predicted concentrations calculated from columns 3 and 5. Column 7 represents experimentally observed concentration by filtering a known volume of the final centrifuged dispersion after drying at 80 °C for 12 h and measuring the final mass. Column 8 represents the amount of mass loss due to removal of an unreacted precursor, calculated from columns 6 and 7.

Focusing on the most viscous product, (20 g sediment per 20 g water or ≈150 g/L)—henceforth referred to as 1DL ink—the processing was further optimized. Since the ink is extremely viscous, similar to an industrial caulk, the best mixing method is to use a high shear mixer (such as a FlackTek Speedmixer) to combine the sediment and water. It is most effective to slowly add the water to the sediment in multiple batches while mixing the solution between each addition. Once combined (Figure 1E), the mixture can be spooned into a centrifuge tube and centrifuged to ensure complete waste separation (Figure S3). As a point of warning, the ink is highly basic (pH ≈ 10) due to the uptake of protons by the 1DL during hydration and should be handled with care. It also has a distinct fishy scent from trimethylamine or TMA^+^ due to the high concentration of 1DL in the dispersion. Once made, however, the ink is highly stable and will remain stable for months without noticeable changes in properties or structure, most likely a consequence of its high zeta potential (−85 mV @ pH 10).^37^ The stability of the ink is best demonstrated by the fact that the ink was prepared at Drexel University prior to shipment to Auburn University for printing. The structural stability is referenced by the Raman spectrum in Figure S4 that is consistent with fresh 1DLs, discussed in detail in the work by Badr et al.^18,38^

Previous studies have shown that even much lower concentrations of 1DL dispersions exhibit lyotropic liquid crystalline, LC, phase behavior.^22^ Under polarized light, the LC phase exhibits birefringence, resulting in two distinct refractive indices that produce varying colors and textures, depending on the thicknesses and degree of orientation. In previous work, the observance of Schlieren textures in the studied biphasic region of 10–40 g/L concentration provided confirmation of the formation of a nematic LC phase.^22^ Higher concentrations have not been studied until now. Under a cross-polarized optical microscopy (POM) the 1DL ink exhibits the bright watercolor-like textures indicative of a nematic gel (Figure 2).

**Figure 2:**
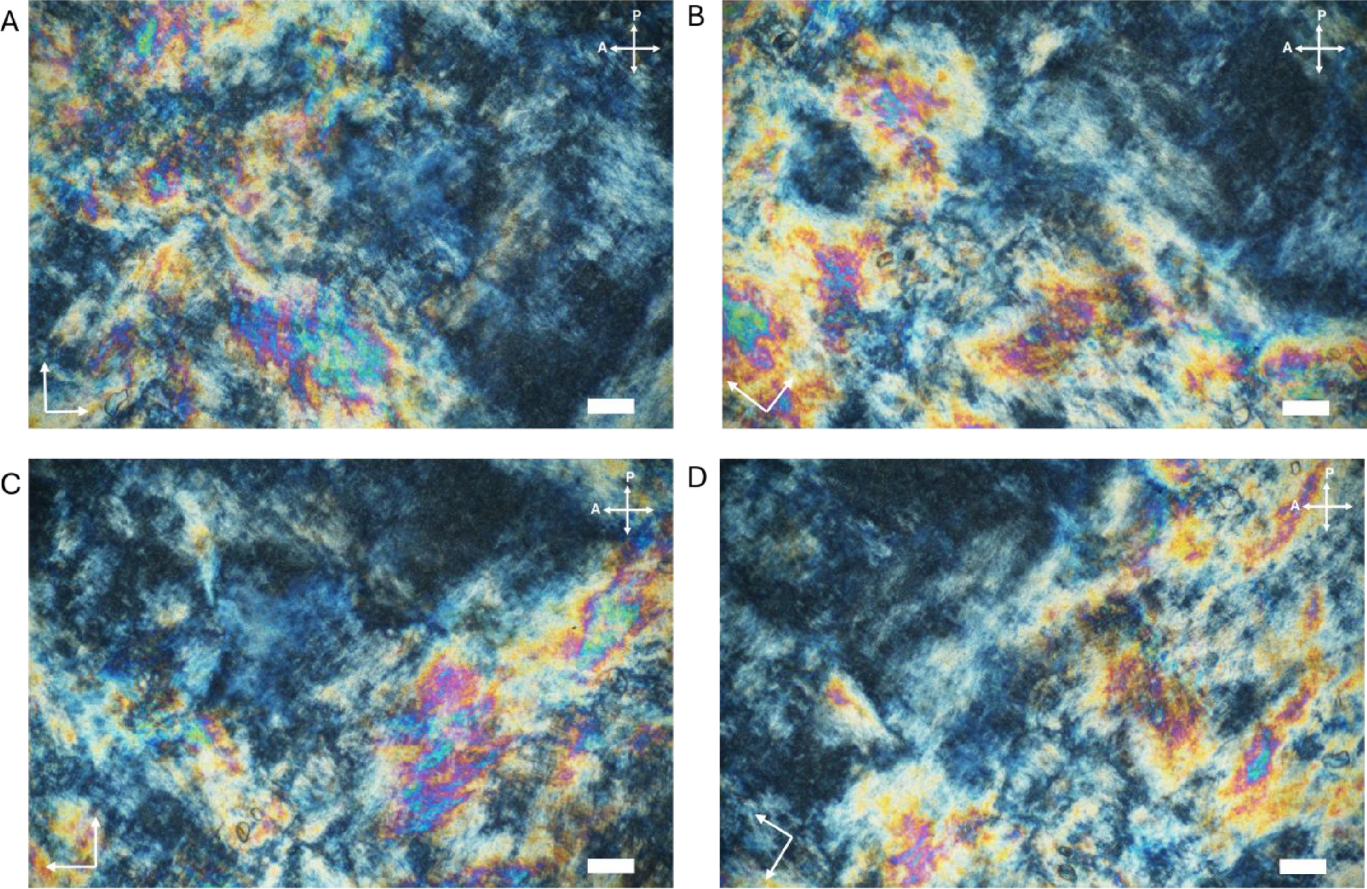
Cross-polarized optical microscopy images of the 1DL ink (nematic gel). At, (A) 0°, (B) 45°, (C) 90°, and (D) 135° stage rotation. Scale bars are 50 μm.

The qualitative suggestion of gel formation was promising for printability as DIW inks require the high yield stress usually associated with colloidal gels. The 1DL ink yield stress and viscosity were estimated by using a benchtop robotic dispenser (Fisnar F4200N) as a capillary rheometer (Figure 3A) and applying the Weissenberg–Rabinowitsch–Mooney (WRM) relations^39^ in accordance with eqs 1–3

$$\tau_{w}=\frac{\Delta PD}{2L}$$ 1

$$\gamma {˙}_{a}=\frac{4Q}{\pi R^{3}}$$ 2

$${\mathrm{\gamma ̇}}_{w}=\frac{{\mathrm{\gamma ̇}}_{a}}{4}\left(3+\frac{d\ln{\mathrm{\gamma ̇}}_{a}}{d\ln\tau_{w}}\right)$$ 3

Here, the wall shear stress (τ_w_) was calculated using a nozzle with a length (*L*) of 12.7 mm and a diameter (*D*) of 1.6 mm, under 10 different applied pressure drops (Δ*P*) across the nozzle, as shown in Figure 3D. Measuring the mass of ink extruded per unit time enabled the determination of the volumetric flow rate, *Q,* which was, in turn, used along with the nozzle radius, *R*, to calculate the apparent shear rate, γ̇_a_. The WRM correction,^40^ which accounts for nonparabolic velocity profiles in non-Newtonian fluids where shear rates are higher near the wall, was applied to calculate the effective shear rate (eq 3). The latter was then used to determine the viscosity as the ratio of shear stress to shear rate. The resulting rheological data (Figure 3) shows that the ink was shear thinning with a low shear viscosity on the order of 200,000 Pa·s. Fitting the data to the Herschel–Bulkley equation (eq 4)­

$$\tau =\tau_{y}+K{\mathrm{\gamma ̇}}^{n}$$ 4

where *K* is a consistency index, *n* is a power law exponent, and τ_y_ is a yield stress, resulted in a τ_y_ = 2940 ± 3.0 Pa, *K* = 6,908 ± 0.90 Pa.s^0.36^, and *n* = 0.36 ± 0.1. For an ink to be printable, it must exhibit an appropriate yield stress, shear thinning behavior, and viscoelastic characteristics to maintain its shape after deposition while ensuring sufficient fluidity for interlayer adhesion. These results highlight that in contrast to lower concentration 1DL dispersions, the 1DL ink meets the yield stress and shear thinning requirements for DIW.

**Figure 3:**
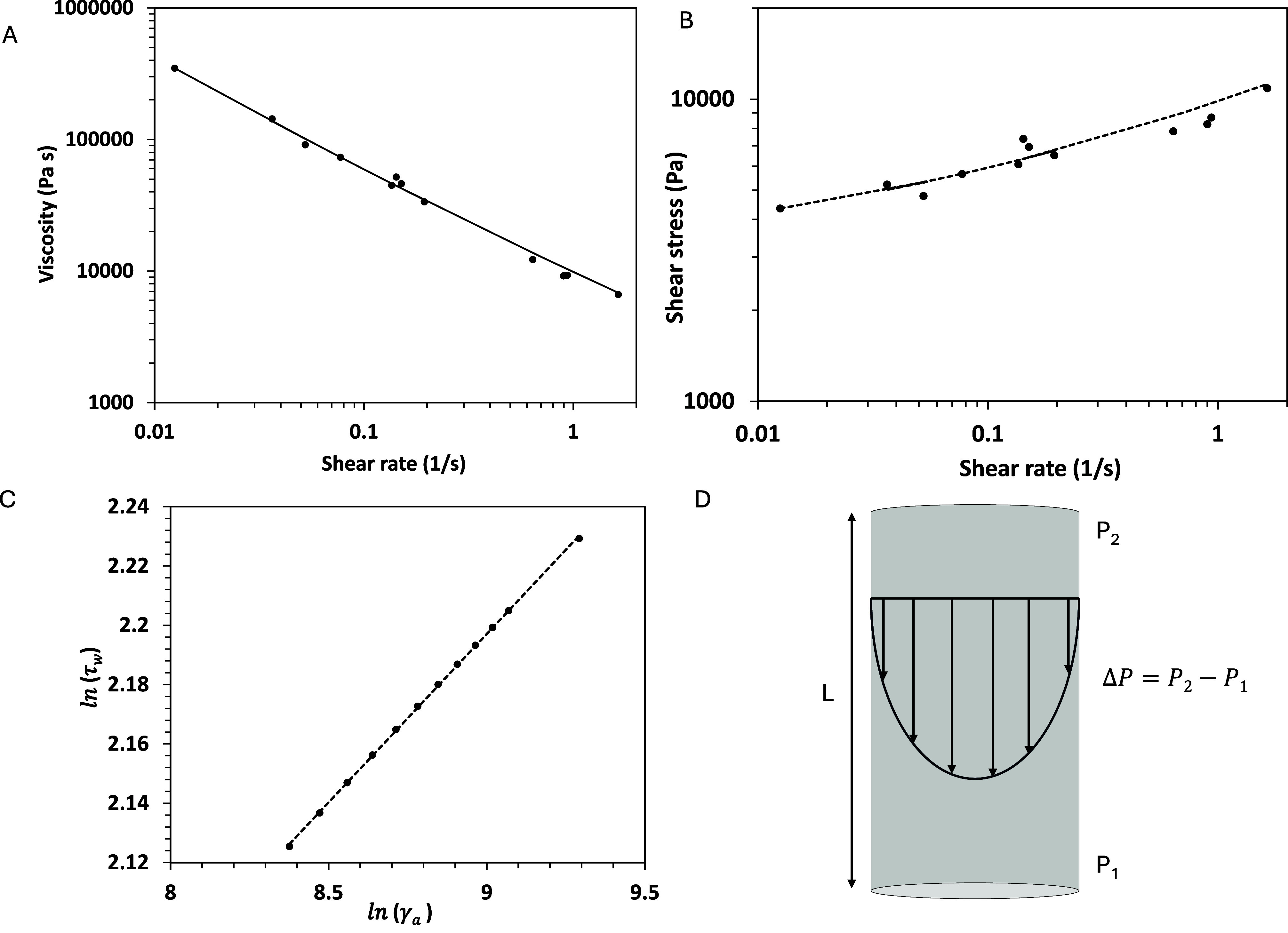
Rheological data of 1DL ink collected using a robotic dispenser. (A) Viscosity vs. shear rate at the wall; (B) shear stress vs. shear rate at the wall; (C) natural log of wall shear stress vs. natural log of apparent shear rate, and (D) schematic of flow through the capillary.

Based on this preliminary assessment of printability, the dispenser was used to produce standard test patterns for evaluating printability, employing nozzle sizes ranging from 0.41 to 1.6 mm in internal diameter and pressures between 68.95 and 275.8 kPa. Figure 4A, Figure 4B, Figure 4C, and Figure 4D show, respectively, the ink being extruded, a four-layer grid pattern, a conical structure, and a filament printed across a gap. All prints were produced using a 0.84 mm diameter nozzle that was 12.7 mm long.

**Figure 4:**
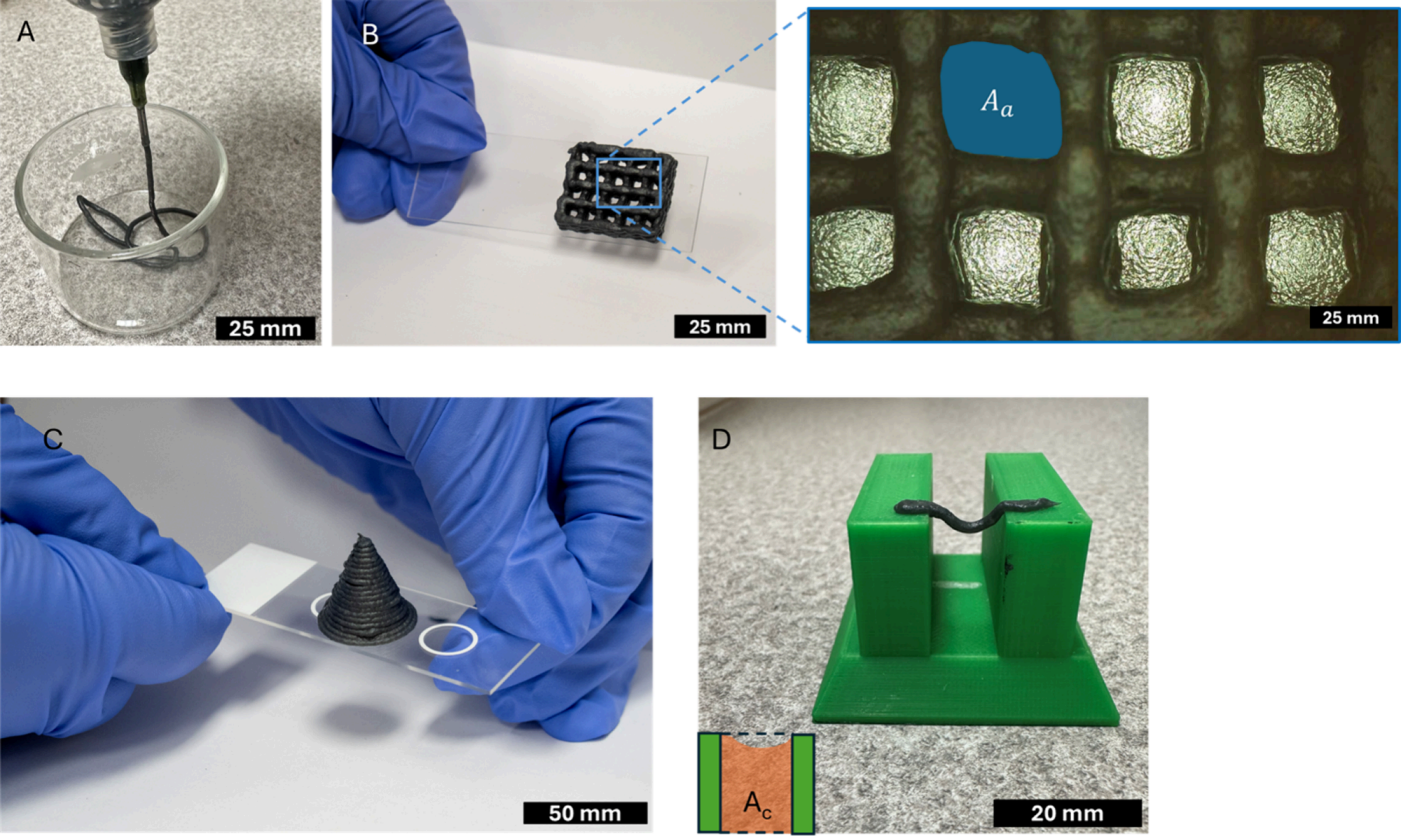
Images of the 1DL prints. (A) Demonstration of filament formation extruded at 137.9 kPa with a 12.7 mm long, 0.84 mm wide nozzle for 5 s, and (B) four-layered printed scaffold. Picture on right is a higher magnification image of area depicted with blue rectangle on left, (C) printed cone with 19 layers and 20 mm diameter and 36° angle, and (D) midspan deflection behavior.

Figure 4B further demonstrates that the print adheres firmly to a glass substrate, maintaining its structure even when tilted without collapsing. Upon air drying, the printed structures exhibited slight shrinkage, which is typical of water-based inks, although their overall structural integrity remained intact. This shrinkage occurs because the ink contains a high solvent fraction, approximately 85 wt % water, and solvent removal during drying typically leads to a reduction in print volume. For instance, the conical structure showed an average linear shrinkage of about 8%. Such shrinkage can be minimized using approaches commonly applied to solvent-based or hydrogel inks, including prefreezing followed by solvent exchange, the use of suitable binders, or postprint curing to enhance structural stability.^41,42^

When immersed in acetic acid, the damp printed structures transformed into a hydrogel, which has been shown to possess good mechanical properties and water stability, as detailed in our previous work.^43^ Figure S5 shows scanning electron microscope (SEM) micrographs of the acetic acid exchanged prints, revealing the wrinkled surface morphology of the dried network. The acetic acid exchanged prints were also characterized with Fourier transform infrared spectroscopy (FTIR) and XRD patterns shown in Figures S6 and S7, respectively the results show that the acid exchange was successful in that the lepidocrocite structure was maintained.

The dimensional accuracy of the prints was analyzed by printing a 30 mm line using a 0.84 mm nozzle of length 12.7 mm (*L*/*D* = 15) at various nozzle speeds (10, 11, 12, and 13 mm/s) and print pressures of 32, 33, and 34 psi corresponding to approximately 220.6, 227.5, and 234.4 kPa, respectively. Print fidelity, which quantifies how well the print width matches the intended width, was evaluated using eq 5

$$f=\frac{l_{\mathrm{ac}}}{l_{\mathrm{th}}}$$ 5

where *l*_ac_ represents the actual average print width measured at 10 different locations, and *l*_th_ is the theoretical width, defined as the nozzle diameter. Print uniformity was assessed as the standard deviation of the print width. The results, summarized in Figure S8, indicate good dimensional precision across various pressure gradients and nozzle sizes. Typically, for most DIW inks, the print width exceeds the nozzle diameter due to a combination of die swelling when leaving the nozzle and insufficient recovery of elasticity prior to deposition. However, at 220.6 and 227.5 kPa, across all studied print speeds, the *l*_ac_’s were smaller than the nozzle diameter (Figure S8A). The reason for this behavior is not currently known but is consistent with a nematic gel and will be studied in more detail in future work. At 227.5 and 234.4 kPa, the print width decreases with increasing print speed (Figure S8A) due to faster elongation of the filament and less time for the ink to accumulate.

Aside from dimensional accuracy, another critical requirement for DIW inks is their ability to be printed into multiple layers without slumping. Figure 4B shows a four-layer grid pattern, and Figure 4C demonstrates the successful printing of a cone (20 mm in diameter and 30 mm high), highlighting the ink’s suitability for fabricating 3D structures with good layer connectivity and no observable slumping. Grid patterns are often used for quantifying printability due to their applicability as scaffolds for tissue engineering, regenerative medicine, drug delivery, energy storage, and microelectronics. The pore factor, also referred to as the printability factor, was determined using eq 6 to assess pore closure of the printed grid.^44^

$$P_{r}=\frac{L^{2}}{16A_{c}}$$ 6

Here, *L* represents the perimeter of the actual pore and *A*_c_ denotes the corresponding pore area, as shown in Figure 4B, where a value of 1.0 indicates a perfectly square pore. The ink demonstrated a printability factor of 0.90 ± 0.01, indicating that it maintains an almost square pore geometry, implying good shape fidelity.

Again referring to Figure 4B, the diffusion rate, *D*_fr_, or rate of material spreading, was calculated using eq 7 on the printed scaffold as detailed in ref 44, which quantifies the percentage deviation of the theoretical area, *A*_t_, from the actual area, *A*_a_, of the grid pores.

$$D_{\mathrm{fr}}=\frac{A_{t}-A_{a}}{A_{t}}\times 100$$ 7

The printed structure exhibited a low material speard of 12% ± 5%. Another important property of DIW inks for printing functional structures is their ability to be printed without a support. To evaluate this, a filament collapse test^44^ was conducted to analyze the midspan deflection of suspended filaments, determining material collapse using the filament collapse factor, *C*_f_, as shown in Figure 4D and eq 8

$$C_{f}=\frac{A_{t}-A_{a}}{A_{t}}\times 100$$ 8

where *A*_t_ represents the theoretical area under the bridge and *A*_a_ represents the actual area under the bridge. If the filament remains stable and forms a straight bridge without collapsing, then the collapse factor is zero. The ink exhibited a low collapse factor for all print conditions tested, exhibiting a good bridging effect as summarized in Table S1, reaching as low as 0.3% at 172.4 kPa pressure and a 30 mm/s print speed.

The results presented herein demonstrate the relative ease for fabricating 1DL suspensions of various concentrations, including a stable, highly concentrated ink (150 g/L) for DIW applications for the first time. The simplicity and scalability of this fabrication process cannot be overstated, requiring only water–no polymer, pH adjustment, surfactants, or dispersants needed. Although printability via DIW was a key focus of this work, ability to produce such a concentrated viscoelastic 1DL dispersion enables other fluid phase manufacturing methods such as fiber spinning and opens a plethora of new directions for 1DL applications development. There is a plethora of further directions available for this development of colloidal nanomaterials.

## Methods

### Materials

The materials utilized in this study were titanium diboride, TiB_2_, (as received, 99.9%, 325 mesh; Thermo Fisher Scientific, Waltham, MA, USA), TMAOH (as received, 25% [w/w] aqueous 99.999%; Alfa Aesar, Ward Hill, MA, USA), EtOH (200 proof; Decon Laboratories, King of Prussia, PA, USA), and acetic acid (Glacial, ACS Reagent, 99.7%).

### 1DL Preparation

Multiple batches of 1DLs were prepared with the following method: 10 g of TiB_2_ was added to 87.5 g of 25 wt % TMAOH aqueous solution in a 250 mL HDPE bottle vented with a single 23-gauge needle. (Warning: The reaction produces significant amounts of H_2_ gas and should not be carried out in closed systems). The bottle was heated and shaken in an incubator (Labnet International Shaking Incubator, NJ, US) at 200 rpm and 80 °C for 4 days. The resulting sediment was combined with EtOH, vortex shaken, and centrifuged (Sorvall ST 16 with 8 × 50 mL Fixed Angle Rotor, Thermo Scientific, Waltham, MA, USA) at 3500 rpm (1500*g*) for 2 min. The clear supernatant was discarded after each wash, and the gray solids at the bottom, referred to throughout this work as 1DL “sediment”, were collected. This was repeated until a pH of ∼7 was achieved (usually 3 times), measured using pH strips.

To form the 1DL suspensions, various water and sediment combinations (Table 1) were mixed via vortex or high shear mixing, as detailed in the main text. The mixture was centrifuged at 10,000 rpm (12,300*g*) for 30 min resulting in highly stable colloidal suspensions while any unreacted precursor settled to the bottom. The concentration of each suspension was estimated by vacuum filtering 2 mL of the suspension through a 25 μm thick microporous monolayer polypropylene membrane (Celgard 3501, Celgard, NC, US) over a fritted glass filter apparatus. Once filtered, the solid was dried in an oven, under vacuum, at 80 °C overnight, and the weight of the residue was measured.

### 1DL Ink Preparation

Focusing on the 1DL ink (≈150 g/L suspension), the process was optimized due to the high viscosity. Twenty grams of 1DL sediment was added to an SC 60 cup (FlackTek Speedmixer, Landrum, SC, USA). Water was added in four increments of 5 mL. After each addition, the cup was mixed using a high shear mixer (DAC 330-100 Pro, FlackTek Speedmixer) at 2,000 rpm for 5 min. The mixture was centrifuged at 10,000 rpm (12,300*g*) for 30 min, resulting in the ink on top of the unreacted precursor if present (Figure S3). The ink was then scooped out of the centrifuge tube and is ready for printing.

### Printing

The 1DL ink was printed by using a benchtop robotic dispenser (Fisnar F4200N). The desired pattern was designed and printed by using its control software. Ink flow was regulated by a pneumatic fluid dispenser (Fisnar DSP501N), ensuring precise deposition onto glass slides as detailed in our previous work.^1^ The print speed was managed via the teach pendant software connected to the dispenser. Some of the 1DL prints were immersed in pure acetic acid for a period of 10 min to promote hydrogel formation.

### Rheology

The rheological measurements were made using the printer as a capillary rheometer, with the capillary length corresponding to a nozzle length of 12.7 mm and the diameter corresponding to a nozzle diameter of 1.6 mm. Viscosity values were determined by evaluating the flow rate at specific pressure gradients and the shear stresses developed at the walls of the nozzle. Each measurement was conducted three times, and the results were averaged over these runs to calculate the relevant parameters using the Weissenberg–Rabinowitsch–Mooney relations,^2^ as detailed in the main text.

### Microscopy

Transmission electron microscope (TEM) micrographs were collected using a field-emission transmission electron microscope (JEOL, Akishima, Tokyo, Japan, JEM2100F). The TEM was operated at 200 keV. Images were collected on a Gatan USC1000CCD camera. Samples were prepared by first diluting the colloidal suspensions to <1 mg/L, then drop casting on a carbon-coated, lacey-carbon, copper TEM grid.

Optical microscopy was performed using a Nikon Eclipse Ni-U microscope in transmission mode with a 20×/0.45 NA LU Plan Fluor and a 60×/1.40 NA oil immersion objective. Images were captured with a digital camera (Nikon DS-Ri2) and Nikon Elements software. To prepare the samples, a drop of the dispersion was transferred onto a clean glass slide by using the tip of a glass rod. A coverslip was then placed on top of the material without the use of a spacer. To prevent solvent loss due to evaporation, the edges of the coverslip were sealed with acetone-based nail polish.^3^

### Material Characterization

FTIR spectra were obtained at a resolution of 4 cm^–1^ in the 400–4000 cm^–1^ range (INVENIO-R with ATR attachment, Bruker Corp., Billerica, MA, USA). The powders were placed directly on the ATR crystal, and pressure was applied by twisting down the hammer. The raw spectra were corrected using a standard ATR correction in Opus software. XRD patterns were acquired with a diffractometer (MiniFlex 600 benchtop XRD, Rigaku, Tokyo, Japan) equipped with a Cu–Kα radiation source scanned from 5 to 65° 2θ with step increments of 0.02 s^–1^ and a 1 s hold time. Raman spectroscopy was performed on a Renishaw inVia Qontor using a 785 nm laser at 1% power, with a 10 s acquisition time and five accumulations, and baseline correction was applied to the resulting spectra.

## Supplementary Material





## Notes

Additional characterization, including TEM, FTIR, XRD, Raman Spectra, and SEM, tables and graphs of print quality data, and an image of 1DL ink after synthesis (PDF)
Video of printing of a cone onto a glass slide (MOV)
