# Supplementary material for: Binder-Free Direct Ink Writing of a Concentrated Dispersion of One-Dimensional Lepidocrocite Titanate Nanofilaments
Source: ACS Nanosci Au. 2025 Nov 18;6(1):102–9. doi: 10.1021/acsnanoscienceau.5c00110 (PMC12921620; doi:10.1021/acsnanoscienceau.5c00110)
Supplement: Supplementary file 1 [file ng5c00110_si_001.pdf]

Supplementary Information for

Binder-Free Direct Ink Writing of a Concentrated Dispersion of 1D Lepidocrocite Titanate Nanofilaments

Francis Mekunye[1], Adam D. Walter[2], Gregory R. Schwenk[2], Michel W. Barsoum[2], Virginia A. Davis,[1]*

[1]Department of Chemical Engineering, Auburn University, 212 Ross Hall, Auburn, Alabama 36830, United States.

[2]Department of Materials Science and Engineering, Drexel University, 3141 Chestnut St, Philadelphia, Pennsylvania 19104, United States.

*Corresponding author

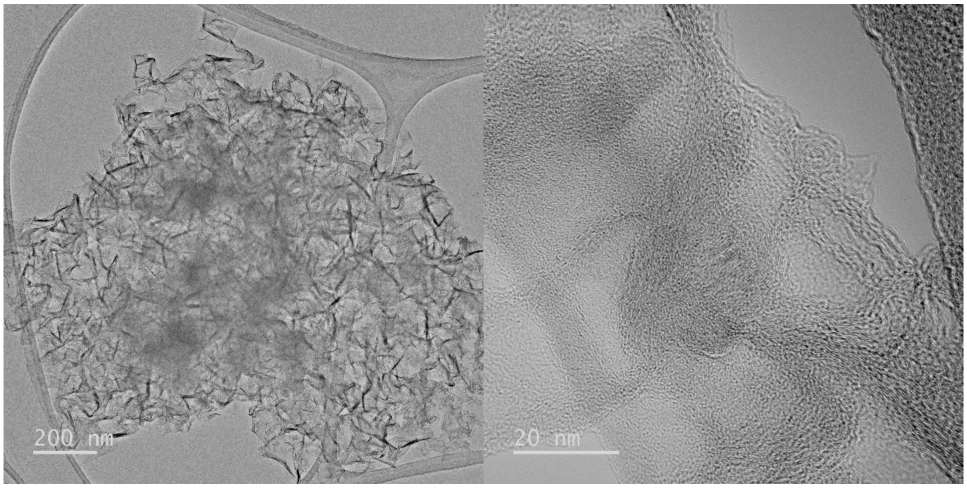

Figure S1. TEM micrographs of dilute 1DL suspension drop cast on grid. Regions of agglomerated 1DLs are visible at low magnification. At high magnification (right), the polymeric-like bundling of 1DLs is visible.

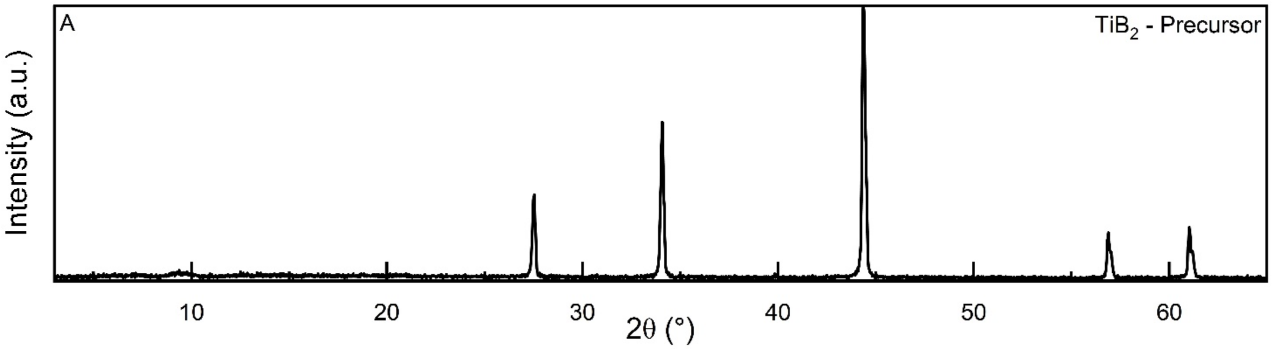

Figure S2. XRD pattern of TiB_2, the precursor used in the 1DL synthesis, detailed in this work.

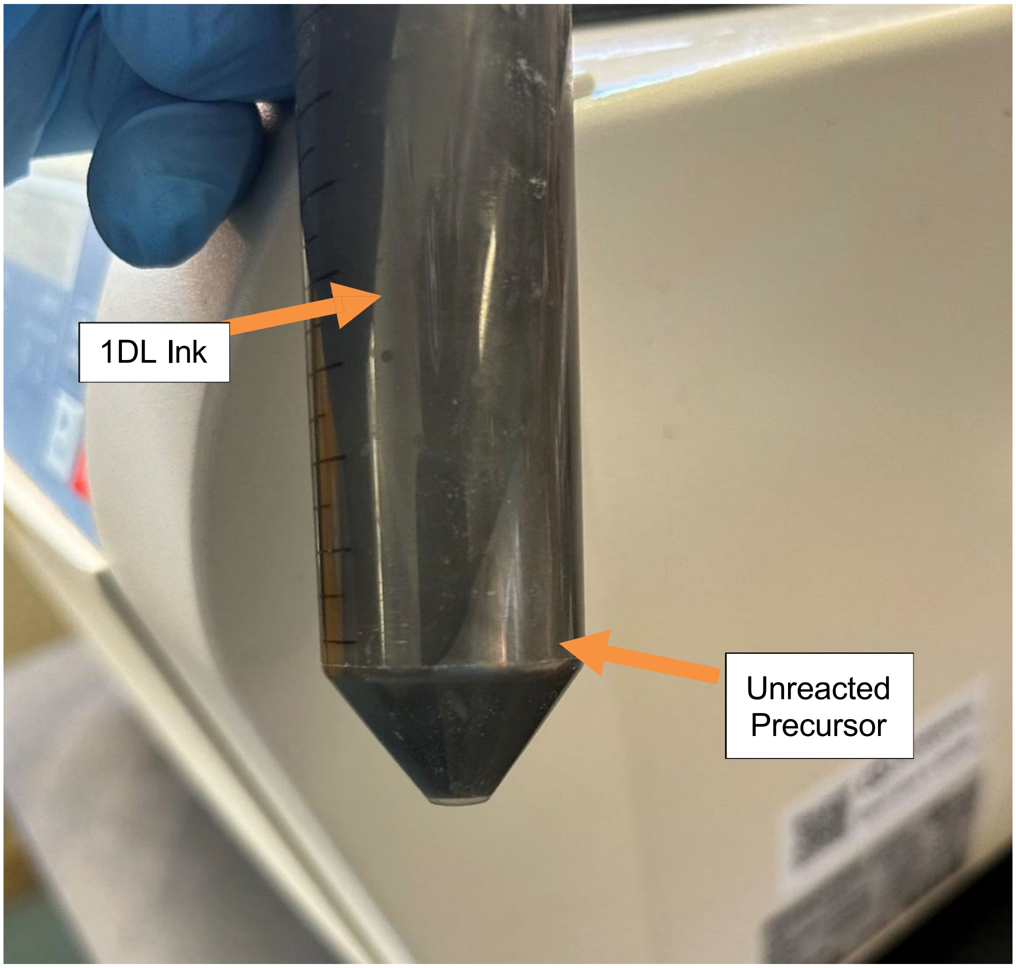

Figure S3. Photograph of 20 g sediment per 20 g water mixture after centrifugation. 1DL ink (concentration ≈ 150 g/L) sits on top of unreacted precursor.

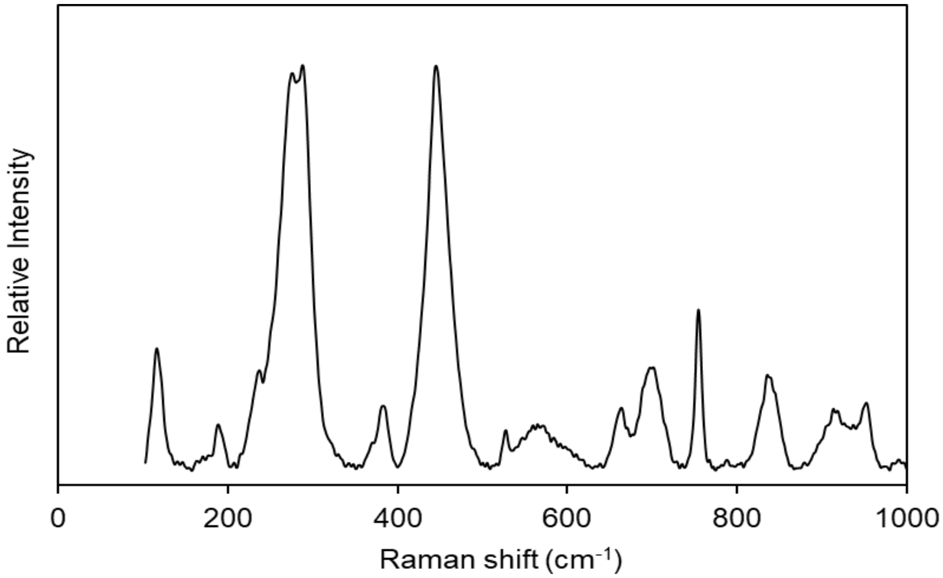

Figure S4. Raman spectrum of 1DL ink after drying, with peaks consistent with the lepidocrocite structure.

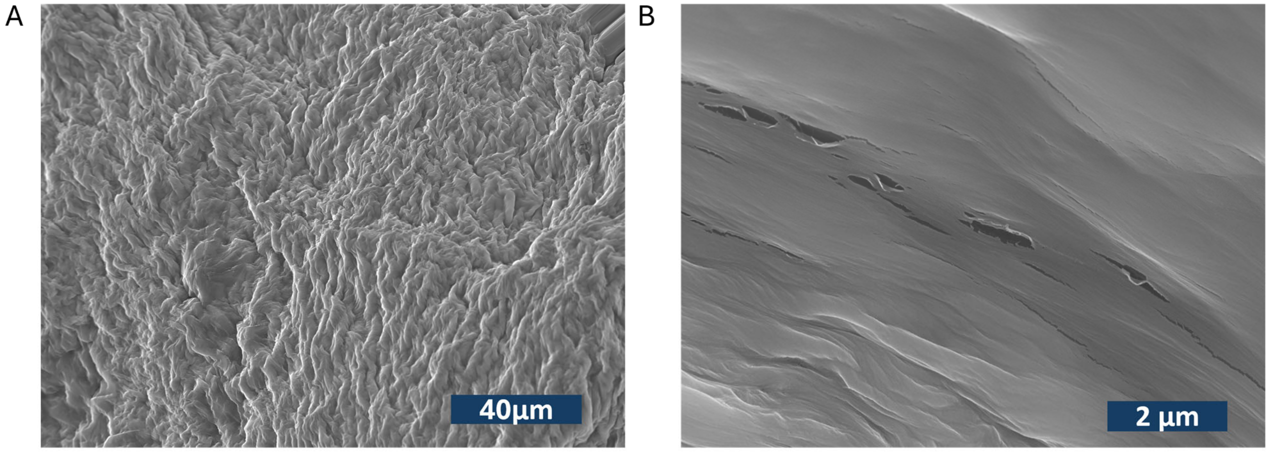

Figure S5. SEM micrographs of dried printed structure, (A) at low magnification showing wrinkled surface morphology of the dried network, and (B) same as A but at a higher magnification.

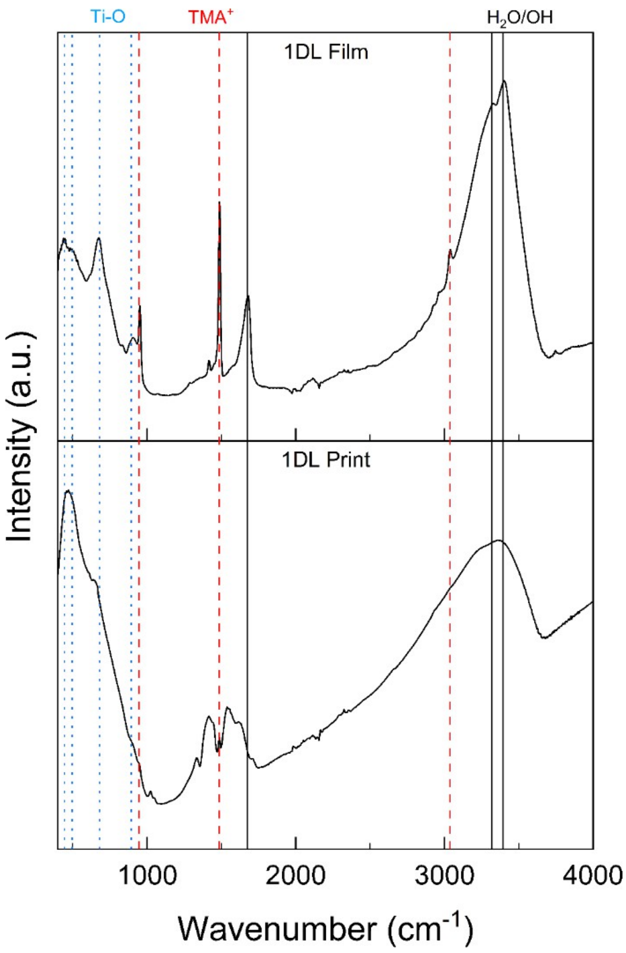

Figure S6. FTIR spectra of standard 1DL film and 1DL print after acetic acid exchange. Note loss of TMA^+ peaks after acid exchange. Samples were finely powdered prior to analysis.

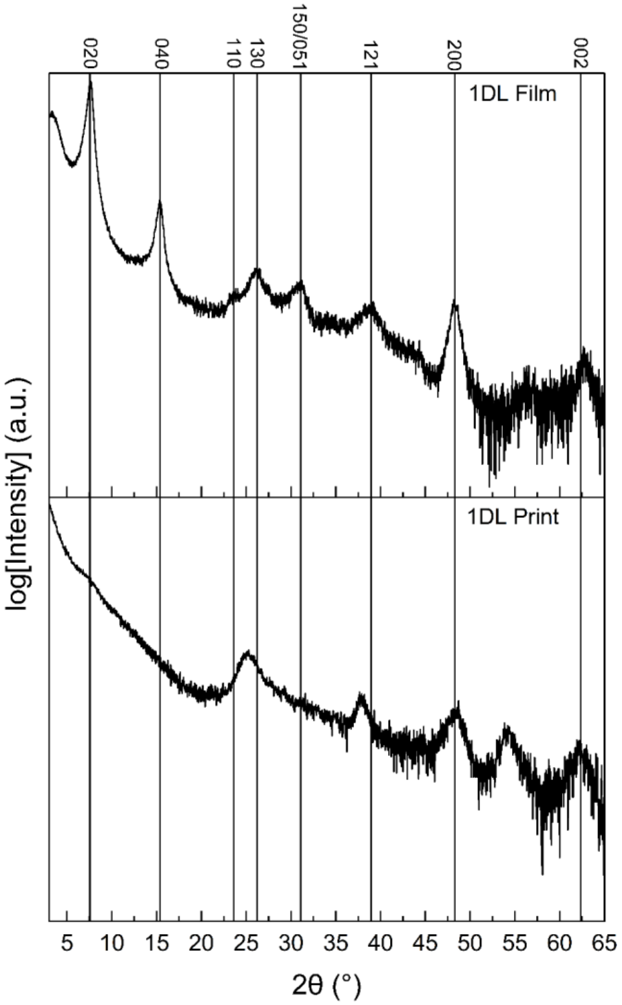

Figure S7. Indexed semilog XRD patterns of standard 1DL filtered film and 1DL print after acetic acid exchange. Indices of peaks are noted on top of figure. Note loss of stacking order (020, 040 peaks) after acid exchange. Samples were finely powdered prior to analysis.

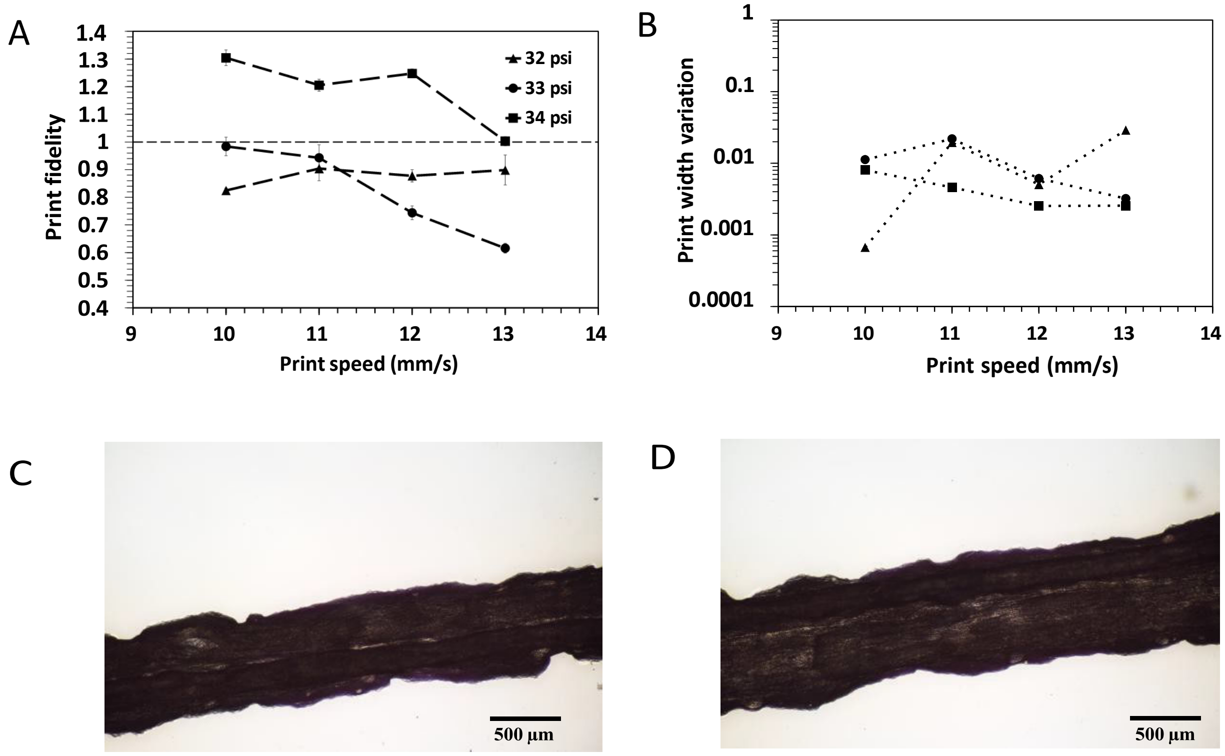

Figure S8. Print quality evaluation. A) Plot of print fidelity vs. print speed for wet ink. B) Plot of print width of variation vs. print speed for wet ink; C) Dried print line at 32 psi (220.6 kPa) at 13 mm/s, D) Same as (C) but at 34 psi (234.4 kPa).

Table S1. Bridging collapse factors at seven different print conditions. Column 4 was calculated from ImageJ by evaluating area under the print line. Column 5 is the designed area between the pillars shown in Figure 4D.

Print Pressure (kPa)	Print Speed (mm/s)	Nozzle Diameter (mm)	Actual Area Under Span (mm^2)	Theoretical Area Under Span (mm^2)	Collapse factor (%)
137.9	10	1.6	139.5	164.85	15
151.7	10	1.6	107.25	164.85	35
151.7	20	1.6	153.58	164.85	7
151.7	30	1.6	163.29	164.85	1
172.4	10	1.6	146.22	164.85	11
172.4	20	1.6	139.28	164.85	16
172.4	30	1.6	164.325	164.85	0.3

REFERENCES

(1) Orangi, J.; Hamade, F.; Davis, V. A.; Beidaghi, M. 3D Printing of Additive-Free 2D Ti3C2Tx (MXene) Ink for Fabrication of Micro-Supercapacitors with Ultra-High Energy Densities. *ACS Nano* **2020**, *14* (1), 640-650. DOI: https://doi.org/10.1021/acsnano.9b07325.

(2) Magnon, E.; Cayeux, E. Precise Method to Estimate the Herschel-Bulkley Parameters from Pipe Rheometer Measurements. *Fluids* **2021**, *6* (4), 157. DOI: https://doi.org/10.3390/fluids6040157.

(3) Woods, M. B.; Beidaghi, M.; Davis, V. A. Phase Behavior and Rheological Properties of Size-Fractionated MXene (Ti3C2Tx) Dispersions. *Langmuir* **2024**, *40* (6), 2907-2917. DOI: https://doi.org/10.1021/acs.langmuir.3c02851.
